# Supplementary material for: Hungry for compliments? Ghrelin is not associated with neural responses to social rewards or their pleasantness
Source: Front Psychiatry. 2023 Apr 3;14:1104305. doi: 10.3389/fpsyt.2023.1104305 (PMC10106620; doi:10.3389/fpsyt.2023.1104305)
Supplement: Supplementary file 1 [file Data_Sheet_1.pdf]

SUPPLEMENTARY MATERIALS

Hungry for compliments? Ghrelin is not associated with neural responses to social rewards or their pleasantness

Uta Sailer, Federica Riva, Jana Lieberz, Daniel Campbell-Meiklejohn, Dirk Scheele, and Daniela M. Pfabigan

Contact information: uta.sailer@medisin.uio.no & daniela.pfabigan@uib.no

The following analyses were inspired by reviewer comments and suggestions. Detailed results can be found in the project repository on the OSF: https://osf.io/tjxvh/

1. Ghrelin variation based on an area-under-the-curve (AUC) approach

An often-used method in endocrinological research is the computation of the area under the curve (AUC) to quantify information from repeated measurements. Based on the recommendations by (1), we computed AUC_g ("area under the curve with respect go ground") with fixed time intervals between ghrelin assessments at T0 and T1 (60 min) and between T1 and T2 (135 min). This calculation required complete ghrelin assessments in both test sessions, which reduced the number of participants available for further analyses though.

Based on AUCg, we probed whether associations (or the lack thereof) between ghrelin concentrations and behavioural and neural measures reported in the main document were also observable with a different quantification of changes in ghrelin concentrations. To this end, we calculated the difference between the liquid-meal and the no-meal test session for AUCg (ΔAUCg) and repeated the correlation analyses of tasks 1 and 2 analogous to those reported in the main document. The correlation between ΔAUCg (after discarding one outlier value) and ΔvmPFC activity in the computer reward outcome phase (win > no-win, task 1) remained significant (r_s=.459, p=.008; N=33). No other correlations with ΔAUCg were significant (all p-values > .131 in task 1 and > .280 in task 2), which mirrors the results obtained with Δghrelin at T1.

2. Exploration of whole brain results with cluster-based FWE correction

In the main document, we pursued an FWE peak-level correction approach to minimize false-positive findings in whole brain analyses. Exploring whole brain activation patterns, we applied an FWE cluster-level correction in both tasks (starting threshold p < .001 uncorrected, FWE-cluster correction p < .05). This resulted in two significant activation clusters that were not detected by a stricter FWE correction.

In the "recognition-by-experts task" (task 1), the contrast liquid-meal > no-meal yielded two significant activation clusters, one in left superior frontal gyrus (-18 -2 54, k=149, $p_{FWE\text{-}corr}$ = .034)

and one in left precentral/postcentral gyrus (-30 -14 60, k=233, $p_{FWE\text{-}corr}$ = .005) in the social reward condition.

3. Exploratory analyses of sex differences in the current study

No sex-specific hypotheses were pre-registered because the planned sample size would not have been large enough. Note, the current sample is not well-balanced regarding the ratio of women to men – about 1/3 of the participants were women. Hence, we recommend interpreting the subsequent results with caution.

We will report exploratory sex-specific analyses below in the same sequence as analyses are reported in the main document. The factor "sex" was added to the respective statistical models – mostly mixed ANOVAs that were followed up by Tukey post-hoc tests. Only participants who described themselves as women or men were included in the subsequent analyses, hence one participant was excluded because they did not want to disclose their sex/gender.

3.1 Manipulation check

Ghrelin concentrations: A main effect of sex and interaction terms of sex x nutritional state, sex x sample time point, and the three-way interaction were added to the linear mixed model testing ghrelin variation during the experiment. In addition to the results in the main document, a main effect of sex (b=426.8, SE=105.4, t(62.3)=4.05, p<.001, semi-partial R^2=0.21) and significant interactions with the factor sex were observed (3-way interaction: b=310.2, SE=90.8, t(222.5)=3.42, p<.001, semi-partial R^2=0.05). In general, women had higher ghrelin concentrations than men, which is repeatedly reported in the literature (e.g., (2)). Descriptively resolving the three-way interaction, it looks as if women displayed a steeper ghrelin suppression caused by the meal than men (right panel in Figure S1, change from T0 to T1). In contrast, in the no-meal condition, only the main effect of sex (with higher concentrations in women than men) was observable – see Figure S1.

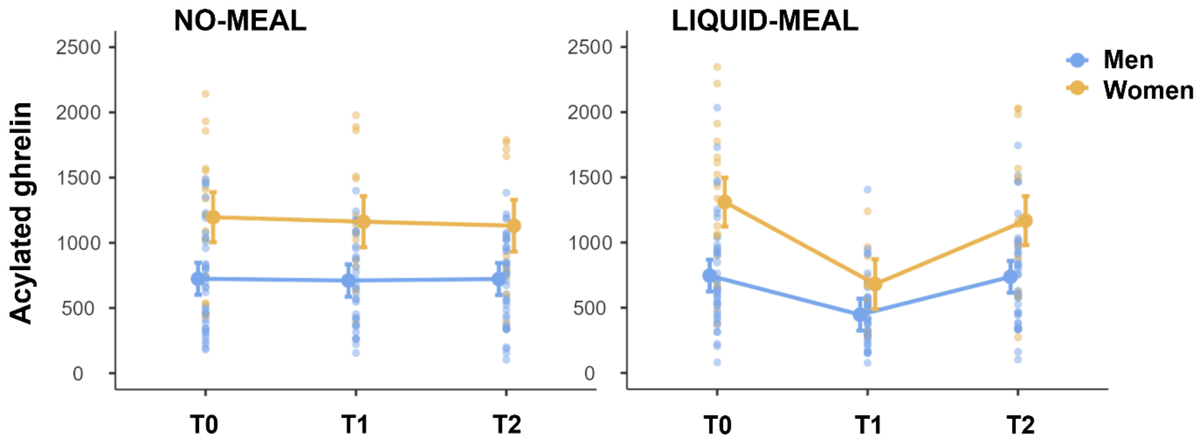

Figure S1. Mean and individual acylated ghrelin concentrations (pg/ml) for the three measurement time points (T0/baseline, T1, T2) separately plotted for women (orange) and men (blue) in the no-meal and the liquid-meal test session. Error bars denote 95% CI.

Blood glucose concentrations at T0: A mixed model ANOVA (nutritional state x sex) resulted in a significant interaction effect ($F(1,57)=6.70$, $p=.012$, $\eta_p^2=.11$). Tukey post-hoc tests showed that blood glucose concentrations in male participants did not differ between the liquid-meal and the no-meal test session ($p=.967$). In contrast, in female participants, blood glucose concentrations assessed at the very beginning of the experiment were higher in the liquid-meal than the no-meal session ($p=.008$).

Bodily and affective states: We computed mixed model ANOVAs (nutritional state x sex) for the sake of comparability with other sex-specific analyses. We acknowledge, however, that the rating data are not normally distributed.

The "Subjective Hunger" ANOVA resulted in a significant interaction effect ($F(1,57)=6.31$, $p=.015$, $\eta_p^2=.10$). Women reported to being less hungry after the meal than men ($p=.023$).

The "Full Stomach" ANOVA resulted in a significant interaction effect ($F(1,57)=4.09$, $p=.048$, $\eta_p^2=.07$). However, post-hoc tests did not show significant sex differences (both p's > .115).

No effects of sex were observed in the "Thirst" ANOVA (all sex-specific p's > .237).

The "Desire to eat" ANOVA resulted in a significant interaction effect ($F(1,57)=5.23$, $p=.026$, $\eta_p^2=.08$). However, post-hoc tests did not show significant sex differences (both p's > .160).

The "Estimated amount one could eat" ANOVA resulted in a significant main effect of sex ($F(1,57)=25.1$, $p<.001$, $\eta_p^2=.31$). Overall, our male participants reported that they could eat a larger amount of food than our female participants.

The mixed ANOVA on the "Composite score" of bodily state ratings resulted in a significant interaction effect ($F(1,57)=8.58$, $p=.005$, $\eta_p^2=.13$), with significant sex differences during the liquid-meal test session (women were overall scoring lower than men).

No effects of sex were observed in the "Willingness to pay for food" ANOVA (all sex-specific p's > .094).

No effects of sex were observed in the "Time since last meal" ANOVA (all sex-specific p's > .913).

No effects of sex were observed for PANAS positive affect (all sex-specific p's > .352) and PANAS negative affect (all sex-specific p's > .323).

Following a Bonferroni correction of the 10 conducted ANOVAs (Bonferroni-corrected p > .005), only the variable "Estimated amount one could eat" showed robust sex differences.

3.2 Task 1

The "Susceptibility index B_{inf}" ANOVA resulted in a significant interaction effect ($F(1,44)=4.26$, $p=.045$, $\eta_p^2=.09$). However, post-hoc tests did not show significant sex differences (both p's > .169).

Adding the between-subjects factor sex to the trust and appreciation ratings of the two experts resulted in significant nutritional state x sex interactions (both p's $< .031$). However, post-hoc tests did not show significant sex differences (all p's $> .218$).

Adding the between-subjects factor sex to the ROI ANOVA models (Bonferroni-corrected $p < .017$) did not result in any sex-specific differences in brain activation in ventromedial prefrontal cortex, or right and left ventral striatum (all p's $> .087$).

3.3 Task 2

Adding the factor sex as main effect to both linear mixed models testing the effects of nutritional state and ghrelin concentrations on subjective pleasantness ratings did not result in significant sex-specific effects (both p's $> .292$). Adding interaction terms with the factor sex decreased model fit and did not result in significant sex-specific results either.

Adding the between-subjects factor sex to the ROI ANOVA models (Bonferroni-corrected $p < .017$) did not result in any sex-specific differences in brain activation in ventromedial prefrontal cortex, or right and left ventral striatum (all p's $> .049$).

3.4 Summary of the sex-specific analyses

Overall, these exploratory analyses did not result in strong support for sex differences in the current study. Here, reward responses to social and nonsocial rewards and their potential interaction with the hormone ghrelin seem to be mostly independent of participants' sex.

References

1. Pruessner JC, Kirschbaum C, Meinlschmid G, Hellhammer DH. Two formulas for computation of the area under the curve represent measures of total hormone concentration versus time-dependent change. Psychoneuroendocrinology. 2003 Oct;28(7):916–31.

2. Makovey J, Naganathan V, Seibel M, Sambrook P. Gender differences in plasma ghrelin and its relations to body composition and bone - an opposite-sex twin study. Clin Endocrinol (Oxf). 2007 Apr;66(4):530–7.
